# Supplementary material for: Does willpower mindset really moderate the ego-depletion effect? A preregistered replication of Job, Dweck, and Walton (2010)
Source: PLoS One. 2023 Jun 29;18(6):e0287911. doi: 10.1371/journal.pone.0287911 (PMC10310002; doi:10.1371/journal.pone.0287911)
Supplement: S1 Appendix — (PDF) [file pone.0287911.s001.pdf]

Visual Illustrations for the Stroop Data Used for the Fixed-Effects Modeling

As noted in the main text, our planned (preregistered) analyses of the main Stroop data (both error rates and RT data) are based on the mean scores calculated for each trial type (i.e., congruent and incongruent in Block 1 and neutral and incongruent in Block 2) for each participant. Although Table 1 in the main article provides all the condition means and *SD*s, it is often helpful to see the distributions of the data points visually. For this reason, we provide here the “rain cloud” plots of the main Stroop data for both types of dependent measures (error rates and log RTs) for each block (Blocks 1 & 2). Because there was one extreme outlier for the error data for Block 1, we are providing two rain cloud plots for this dataset, one with and one without the extreme outlier.
Here is the summary of the five rain cloud plots presented in this section:

- **Figure A**: Block 1 error data with all the data points (including one extreme outlier)
- **Figure B**: Block 1 error data without one extreme outlier and Block 2 error data
- **Figure C**: Block 1 and Block 2 log RT data

As is clear from Figure A, there was one extreme outlier in the control condition, whose error rate for Block 1 was over 60% (61.5%), well above the next highest error rates observed in either of the two conditions (control or depletion). As mentioned in the results section of the main article, this one extreme outlier had some noticeable impact on the statistical results, even though our sample size was almost three times as many as that for Job et al.’s original study.

Figure A. Rain cloud plot for the error rate distribution for each participant for Block 1 .

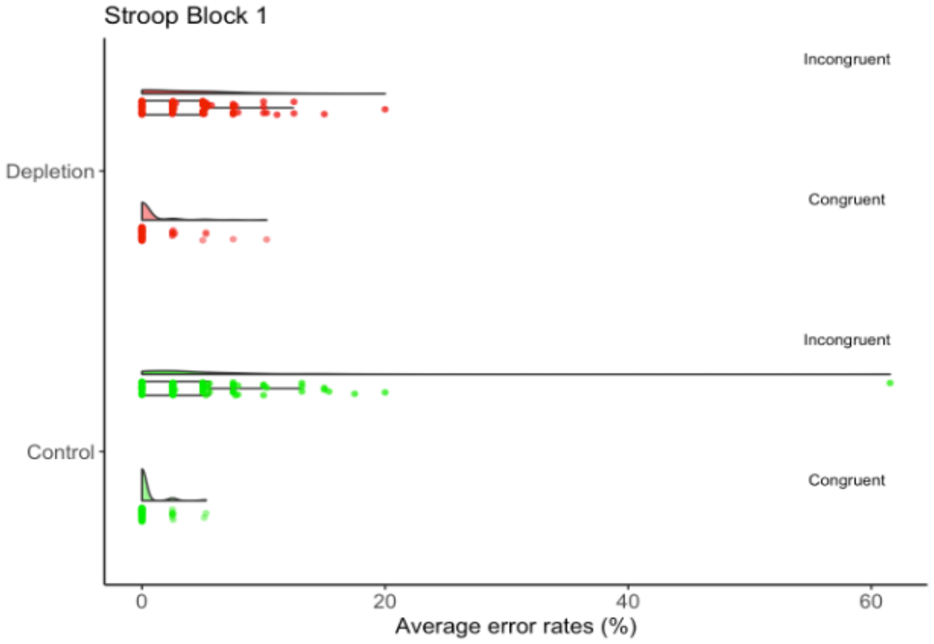

As shown in Figure B (the outlier from Figure A is removed), the error data demonstrate a clear general tendency for the floor effect for both Blocks 1 and 2, because the error rates were low in our study. Arcsine-transformed accuracy data showed better distributional characteristics, but this transformation was not able to turn this skewed distribution into a normal distribution (for the results of the analyses based on arcsine-transformed Stroop accuracy data, see Table A below).

Figure B. Rain cloud plot for the error rate distribution for each participant for Block 1 (left panel) and Block 2 (right panel).

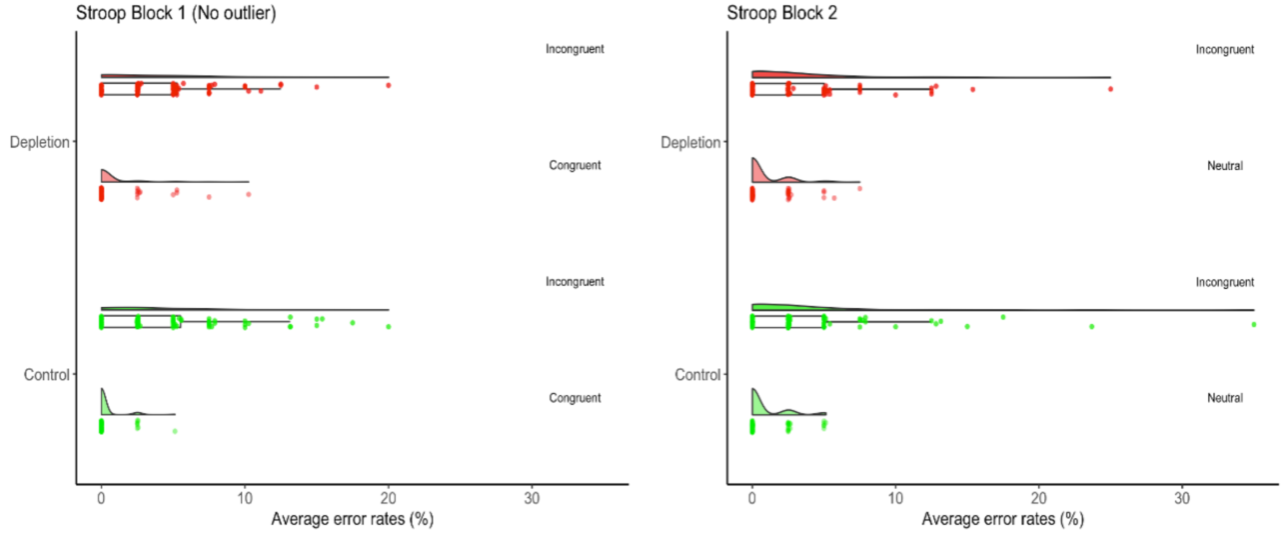

In contrast, the log RT data are more normally distributed for both blocks (Figure C), less skewed than the raw RT data we also analyzed (for the results of the fixed-effects and mixed-effects analyses based on raw RT data, see Table A and Table B).

Figure C. Rain cloud plot for the log RT distribution for each participant for Block 1 (left panel) and Block 2 (right panel).

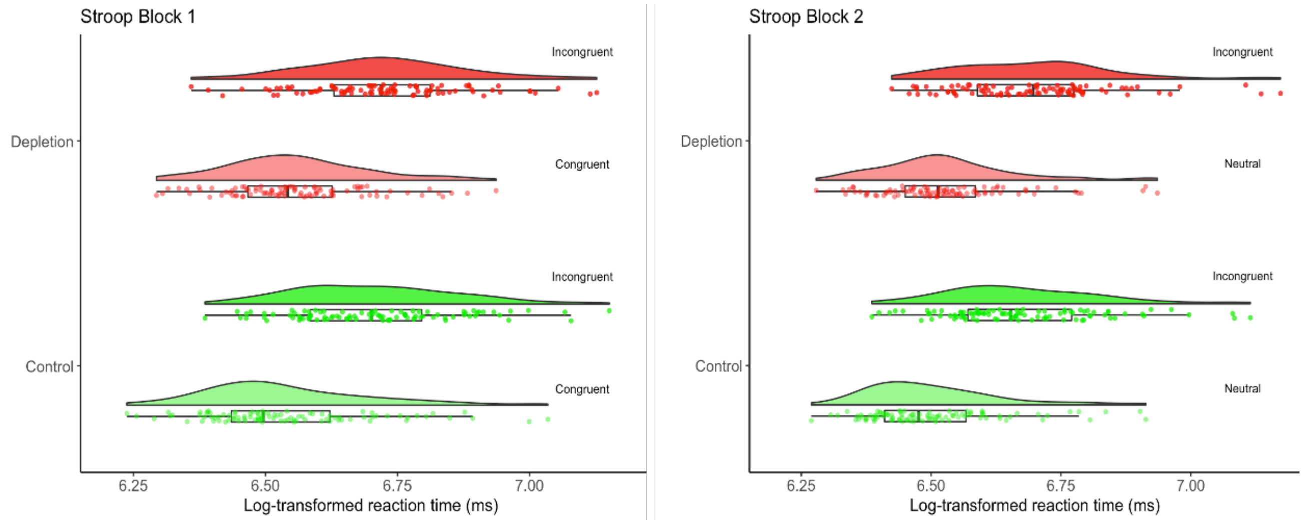

Fixed-Effects and Mixed-Effects Modeling Results Based on Arcsine-Transformed Accuracy Data and Raw RT Data

This section provides the results of our supplementary (but preregistered) analyses based on arcsine-transformed accuracy data and raw RT data for both Stroop Block 1 and Block 2. (Note that Arcsine transformation has to be applied to the proportion correct measure, rather than the error rate or proportion incorrect measure).

We conducted the same fixed-effects and mixed-effects analyses reported in the main text, but with different dependent measures (see Table A for the results of the fixed-effects analyses and Table B for the mixed-effects analyses). The purpose of these additional analyses was to make sure that the results we report are robust to different data transformations. Indeed, the results based on the arcsine-transformed accuracy data and the raw RT data are consistent with those based on the raw error and log RT data reported in the main article (see Tables 4 and 5 in the main article for comparison).

Table A. Results from fixed-effect regression models using arcsine-transformed accuracy rates and raw RTs.

	**Arcsine-transformed accuracy**				**Raw RTs**			
	B	*SE*	*CI*	*p*	*B*	*SE*	*CI*	*p*
**Block 1**								
Intercept	-0.139	0.010	[-0.158, -0.120]	**<0.001**	134.467	4.823	[124.952, 143.983]	**<0.001**
Condition (C)	0.017	0.010	[-0.003, 0.036]	0.090	1.191	4.823	[-8.324, 10.707]	0.805
Mindset (M)	-0.001	0.010	[-0.020, 0.019]	0.942	-2.774	4.835	[-12.314, 6.766]	0.567
C x M	-0.014	0.010	[-0.033, 0.006]	0.171	-1.016	4.835	[-10.557, 8.524]	0.834
**Block 2**								
Intercept	-0.092	0.010	[-0.112, -0.072]	**<0.001**	134.505	4.561	[125.507, 143.504]	**<0.001**
Condition (C)	0.005	0.010	[-0.015, 0.025]	0.599	0.420	4.561	[-8.578, 9.419]	0.927
Mindset (M)	0.017	0.010	[-0.003, 0.037]	0.100	-2.019	4.573	[-11.041, 7.003]	0.659
C x M	-0.003	0.010	[-0.023, 0.017]	0.757	-2.543	4.573	[-11.564, 6.479]	0.579

Note. Stroop effect (arcsine accuracy) was computed as the arcsine-transformed proportion of correct responses in incongruent trials minus the arcsine-transformed proportion of correct responses in congruent trials (Block 1) or asterisk trials (Block 2). *B* = unstandardized regression parameters. *SE* = standard errors for *B*. *CI* = 95% confidence-intervals for *B* estimates. Condition (between-subjects) was coded as -1 for the control condition and 1 for the depletion condition. Mindset = the beliefs about strenuous mental activity subscale score (between-subjects), which was mean-centered and standardized.

Note that Table B, which presents the results of the mixed-effects analyses, includes only the raw RT data (i.e., no analysis of the arcsine-transformed accuracy data). This is because the mixed-effects analyses of the error data we reported in Table 5 (the left panel) in the main article were logistic-regression analyses focusing on the binary outcomes of the individual trials (correct or incorrect). Given that arcsine transformation must be applied to the proportion correct measure (not at the level of

individual trials), it was not possible to conduct mixed-effects analyses for the arcsine-transformed accuracy data. This was the main reason for our decision to report the results of the raw error data in the main article, even though arcsine transformation helps spread out the scores in the high-accuracy range and generally improves the distributional characteristics of the accuracy data.

Table B. Results from mixed-effect regression models using raw RTs.

	**Raw RTs**			
Predictors	*B*	*SE*	*CI*	*p*
**Block 1**				
Intercept	785.224	9.894	[765.832, 804.616]	<**0.001**
Condition (C)	7.325	8.923	[-10.164, 24.814]	0.412
Mindset (M)	-20.467	8.947	[-38.003, -2.931]	**0.022**
Trial Type (T)	67.377	4.909	[57.757, 76.998]	<**0.001**
C x M	-3.141	8.947	[-20.677, 14.395]	0.726
C x T	0.607	2.413	[-4.124, 5.337]	0.802
M x T	-1.298	2.423	[-6.048, 3.452]	0.592
C x M x T	-0.493	2.423	[-5.243, 4.257]	0.839
**Block 2**				
Intercept	759.009	9.168	[741.041, 776.977]	<**0.001**
Condition (C)	8.824	8.251	[-7.348, 24.996]	0.285
Mindset (M)	-12.471	8.273	[-28.686, 3.743]	0.132
Trial Type (T)	67.356	4.604	[58.333, 76.379]	<**0.001**
C x M	-7.861	8.273	[-24.075, 8.354]	0.342
C x T	0.263	2.287	[-4.220, 4.746]	0.908
M x T	-1.085	2.295	[-5.583, 3.414]	0.636
C x M x T	-1.192	2.295	[-5.691, 3.306]	0.603

Note. B = unstandardized regression parameters. *OR* = unstandardized odds ratios. *SE* = standard errors for *OR* and *B. CI* = 95% confidence-intervals for *B* estimates. Condition (between-subjects) was coded as -1 for the control condition and 1 for the depletion condition. Mindset = the beliefs about strenuous mental activity subscale score (between-subjects), which was mean-centered and standardized. Trial type was coded as (-1 for congruent trials in Block 1 and neutral trials in Block 2 and 1 for incongruent trials in both Blocks 1 and 2). *P*-values are calculated based on Satterthwate's approximations.

Results of the Error and Log RT Analyses Focusing on Only the 1st Subblock of the Main Stroop Block 1 (the Replication Block)

As noted in the method section, Block 1 of the main Stroop task was intended to be the direct replication block. Because we increased the number of trials substantially (40 congruent and 40 incongruent trials in Block 1 of our study vs. 24 congruent and 24 incongruent trials in the original study by JDW2010), one could argue that, perhaps due to decay or dissipation of the depletion effect, the hypothesized effects of ego-depletion and its moderation by willpower mindset might be observed clearly if the analysis focused on the early portion of the Block 1 Stroop trials. Because our Block 1 Stroop trials were administered in two subblocks of 40 trials each, we conducted the same fixed-effects and mixed-effects analyses just for the initial subblock of the Block 1 Stroop trials (a total of 40 trials, which is comparable to the total number of Stroop trials administered in the original study [48]).

The relevant condition means (both the first and second subblocks of Block 1) are summarized in Table C below. As shown in the table, there was no evidence for the larger number of Stroop trials in this study contributed to the absence of the overall ego-depletion effect or its moderation by willpower mindset. The results of the fixed-effects analyses for the first subblock only are summarized in Table D, and the results of the corresponding mixed-effects analyses are summarized in Table E.

Table C. Descriptive statistics for Stroop performance measures for the first subblock of Stroop Block 1.

	Control			Depletion				
	n	***M***	***SD***	***n***	***M***	***SD***	***t***	***p***
Error rate for congruent trials (%)	95	0.38	1.74	92	0.39	1.54	0.016	0.987
Log RT for congruent trials	95	6.54	0.16	92	6.57	0.14	1.261	0.209
Error rate for incongruent trials (%)	95	4.31	7.57	92	2.95	4.28	-1.506	0.134
Log RT for incongruent trials	95	6.70	0.16	92	6.72	0.15	0.799	0.425
Difference in error rates (Inc - Cong)	95	3.93	7.00	92	2.56	4.62	-1.567	0.119
Difference in Log RT (Inc - Cong)	95	0.16	0.08	92	0.15	0.08	-0.757	0.450

Note. The *t*-values and *p*-values are results from independent-sample *t*-tests comparing differences between participants in the control condition and participants in the depletion condition.

Table D. Results from fixed-effect regression models for the first subblock of Stroop Block 1.

	Stroop Interference (Error)				Stroop Interference (Log RT)			
	B	*SE*	*CI*	*p*	*B*	*SE*	*CI*	*p*
Intercept	3.228	0.435	[2.369, 4.087]	**<0.001**	0.155	0.006	[0.143, 0.166]	**<0.001**
Condition (C)	-0.679	0.435	[-1.538, 0.180]	0.121	-0.004	0.006	[-0.016, 0.007]	0.459
Mindset (M)	-0.131	0.437	[-0.993, 0.730]	0.764	-0.002	0.006	[-0.014, 0.010]	0.714
C x M	0.597	0.437	[-0.264, 1.458]	0.173	-0.002	0.006	[-0.013, 0.010]	0.789

Note. B = Regression parameters, not standardized with respect to the DV. *SE* = Standard errors for *B*. *CI* = 95% confidence-intervals for *B* estimates. Condition (between-subjects) was coded as -1 for the control condition and 1 for the depletion condition. Mindset = the beliefs about strenuous mental activity subscale score (between-subjects), which was mean-centered and standardized.

Table E. Results from mixed-effect regression models for the first subblock of Stroop Block 1.

	Odds of Incorrect Response				Log RT			
Predictors	*OR*	*SE*	*CI*	*p*	*B*	*SE*	*CI*	*p*
Intercept	0.001	0.711	[0.000, 0.004]	**<0.001**	6.630	0.013	[6.605, 6.654]	**<0.001**
Condition (C)	0.952	0.337	[0.491, 1.842]	0.883	0.012	0.011	[-0.009, 0.033]	0.252
Mindset (M)	1.196	0.318	[0.642, 2.229]	0.573	-0.029	0.011	[-0.049, -0.008]	**0.007**
Trial Type (T)	23.822	0.706	[5.971, 95.033]	**<0.001**	0.078	0.008	[0.063, 0.092]	**<0.001**
C x M	1.105	0.317	[0.594, 2.057]	0.752	-0.005	0.011	[-0.025, 0.016]	0.669
C x T	0.879	0.332	[0.458, 1.686]	0.698	-0.002	0.003	[-0.008, 0.004]	0.462
M x T	0.897	0.312	[0.486, 1.654]	0.727	-0.001	0.003	[-0.007, 0.005]	0.767
C x M x T	1.055	0.312	[0.573, 1.944]	0.863	-0.001	0.003	[-0.007, 0.005]	0.792

Note. B = regression parameters, not standardized with respect to the dependent variable. *OR* = unstandardized odds ratios. *SE* = standard errors for *OR* and *B*. *CI* = 95% confidence-intervals for *B* estimates. Condition (between-subjects) was coded as -1 for the control condition and 1 for the depletion condition. Mindset = the beliefs about strenuous mental activity subscale score (between-subjects), which was mean-centered and standardized. Trial type was coded as (-1 for the congruent trials and 1 for the incongruent trials). *P*-values for linear mixed effects models are calculated by lme4 package in R, based on Satterthwaite's approximations to degrees of freedom. *P*-values for binomial logistic mixed effects models are calculated using Wald's *Z* statistic as estimated by the lme4 package in R.

Results of Secondary Analyses with the Trait Self-Control Measure

In this section, we present the results of both fixed-effects and mixed-effects analyses we conducted for the trait self-control measure (we used the same models, but, instead of willpower mindset, we included trait self-control). Table F reports the results of the fixed-effects analyses, and Table G reports the results of mixed-effects analyses. The descriptive statistics for these questionnaire measures are provided in Table A above. As is clear from both Table F and G, there was no evidence for either the main effect of trait self-control on Stroop performance or the moderation of the ego-depletion effect by trait self-control.

Table F. Results from fixed-effect regression models using trait self-control as the target moderating variable.

	**Stroop Interference (Error)**				**Stroop Interference (Log RT)**			
	B	*SE*	*CI*	*p*	*B*	*SE*	*CI*	*p*
**Block 1**								
Intercept	3.794	0.421	[2.963, 4.624]	<**0.001**	0.166	0.005	[0.155, 0.177]	<**0.001**
Condition (C)	-0.691	0.421	[-1.522, 0.139]	0.102	-0.002	0.005	[-0.013, 0.009]	0.722
TSC	0.408	0.422	[-0.425, 1.241]	0.335	-0.001	0.005	[-0.011, 0.010]	0.919
C x TSC	0.032	0.422	[-0.800, 0.865]	0.939	0.003	0.005	[-0.007, 0.014]	0.539
**Block 2**								
Intercept	2.524	0.353	[1.828, 3.220]	<**0.001**	0.170	0.005	[0.160, 0.179]	<**0.001**
Condition (C)	-0.252	0.353	[-0.948, 0.444]	0.476	-0.002	0.005	[-0.011, 0.008]	0.723
TSC	-0.044	0.354	[-0.742, 0.654]	0.902	-0.002	0.005	[-0.011, 0.008]	0.756
C x TSC	0.090	0.354	[-0.608, 0.788]	0.799	-0.004	0.005	[-0.013, 0.006]	0.460

Note. B = unstandardized regression parameters. *SE* = standard errors for *B*. *CI* = 95% confidence-intervals for *B* estimates. Condition (between-subjects) was coded as -1 for the control condition and 1 for the depletion condition. TSC = the trait self-control scale score (between-subjects), which was mean-centered and standardized.

Table G. Results from mixed-effect regression models using Trait Self-Control as an independent variable.

	Odds of Incorrect Response				Log RT			
Predictors	*B*	*SE*	*CI*	*p*	*B*	*SE*	*CI*	*p*
**Block 1**								
Intercept	0.003	0.496	[0.001, 0.009]	**<0.001**	6.632	0.012	[6.609, 6.656]	**<0.001**
Condition (C)	1.089	0.178	[0.768, 1.543]	0.634	0.009	0.011	[-0.011, 0.030]	0.375
TSC	1.107	0.182	[0.774, 1.582]	0.578	-0.002	0.011	[-0.023, 0.019]	0.858
Trial Type (T)	8.208	0.490	[3.140, 21.459]	**<0.001**	0.083	0.006	[0.071, 0.095]	**<0.001**
C x TSC	1.117	0.183	[0.781, 1.598]	0.544	0.003	0.011	[-0.018, 0.024]	0.765
C x T	0.824	0.164	[0.598, 1.136]	0.237	-0.001	0.003	[-0.006, 0.004]	0.735
TSC x T	1.014	0.168	[0.729, 1.409]	0.936	-0.000	0.003	[-0.006, 0.005]	0.929
C x TSC x T	0.909	0.168	[0.654, 1.265]	0.572	0.002	0.003	[-0.004, 0.007]	0.561
**Block 2**								
Intercept	0.008	0.185	[0.005, 0.011]	**<0.001**	6.601	0.011	[6.579, 6.622]	**<0.001**
Condition (C)	0.981	0.107	[0.796, 1.210]	0.859	0.012	0.010	[-0.008, 0.031]	0.239
TSC	1.011	0.109	[0.817, 1.251]	0.918	0.001	0.010	[-0.018, 0.021]	0.888
Trial Type (T)	2.354	0.175	[1.670, 3.318]	**<0.001**	0.085	0.006	[0.074, 0.096]	**<0.001**
C x TSC	1.027	0.109	[0.830, 1.271]	0.804	0.001	0.010	[-0.018, 0.021]	0.889
C x T	0.962	0.096	[0.797, 1.161]	0.685	-0.001	0.002	[-0.006, 0.004]	0.741
TSC x T	0.994	0.098	[0.820, 1.204]	0.948	-0.001	0.002	[-0.006, 0.004]	0.733
C x TSC x T	0.980	0.098	[0.809, 1.188]	0.840	-0.002	0.002	[-0.007, 0.003]	0.452

Note. B = Unstandardized regression parameters. *OR* = Unstandardized odds ratios. *SE* = Standard errors for *OR* and *B*. *CI* = 95% confidence-intervals for *B* estimates. Condition (between-subjects) was coded as -1 for the control condition and +1 for the depletion condition. TSC = the trait self-control scale score (between-subjects), which was mean-centered and standardized. Trial type was coded as (-1 for the congruent trials in Block 1 and the neutral (asterisk) trials in Block 2 and 1 for the incongruent trials for both Blocks 1 and 2). *P* values for binomial logistic mixed-effects models (for the error data) were calculated using Wald's Z statistic as estimated by the lme4 package in R. *P* values for linear mixed-effects models (for the log RT data) are calculated by lme4 package in R, based on Satterthwaite's approximations to degrees of freedom.
